# Crystal structure of 2-(1,3,7,9-tetra­methyl-2,4,6,8-tetra­oxo-1,2,3,4,6,7,8,9-octa­hydro­pyrido[2,3-*d*:6,5-*d*′]dipyrimidin-5-yl)benzamide di­methyl­formamide hemisolvate

**DOI:** 10.1107/S1600536814020200

**Published:** 2014-09-17

**Authors:** Armen Ayvazyan

**Affiliations:** a Molecule Structure Research Center of, Scientific Technological Center of Organic and Pharmaceutical Chemistry of National Academy of Sciences Republic of Armenia, Azatutyan ave. 26, Yerevan 0014, Armenia

**Keywords:** crystal structure, heterocyclic compounds, amide, hydrogen bonding, tetra­mer

## Abstract

The title compound, C_20_H_18_N_6_O_5_·0.5C_3_H_7_NO, crystallized as a di­methyl­formamide (DMF) solvate. In the main mol­ecule, the dihedral angle between the pyridodi­pyrimidine fused-ring system and the benzamide substituent is 82.26 (11)°. In the crystal, the benzamide mol­ecules are linked by N—H⋯O hydrogen bonds to generate tetra­mers with an approximate square-prismatic shape, which appears to correlate with the tetra­gonal crystal symmetry. The DMF mol­ecule is disordered about a crystallographic twofold axis and accepts a C—H⋯O inter­action from the benzamide mol­ecule.

## Abstract 2

In the crystal, the benzamide mol­ecules are linked by N—H⋯O hydrogen bonds to generate tetra­mers with an approximate square-prismatic shape, which appears to correlate with the tetra­gonal crystal symmetry.

## Chemical context   

Compounds containing fused pyrimidine rings show diverse and inter­esting biological properties. In particular, the representatives of this family show anti­viral (Hossain *et al.*, 1997 ▶), anti­bacterial (Sabnis & Rangnekar, 1990 ▶), anti-AIDS (Joseph & Burke, 1993 ▶) and anti­nociceptive (Bookser *et al.*, 2005 ▶) activities and may serve as non-nucleoside reverse transcriptase inhibitors as well (De Clercq, 1996 ▶). Such a broad spectrum of biological properties for these compounds gives rise to inter­est in their structures and in this paper the structure of the title solvate, (I), is described.
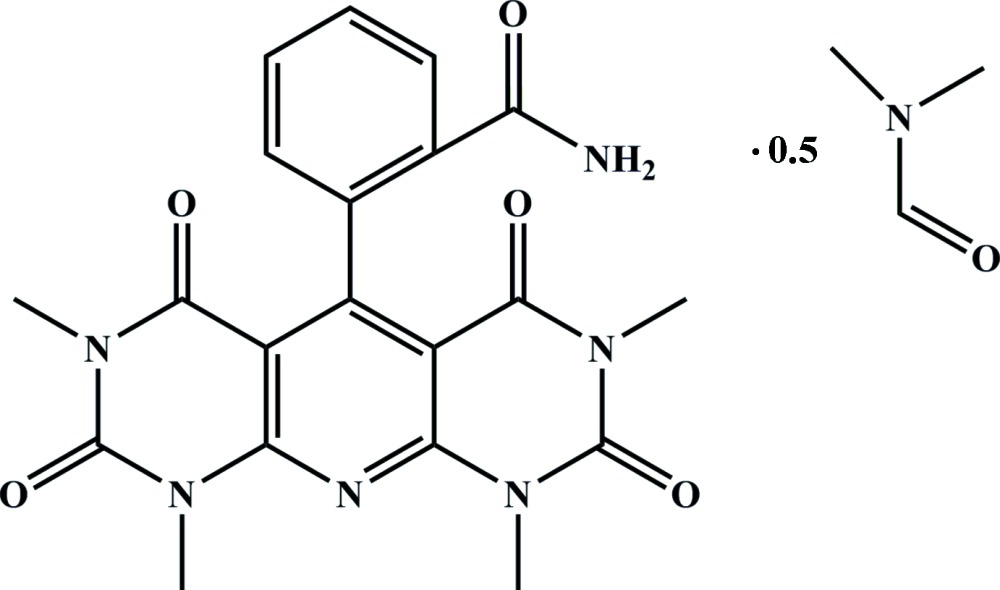


## Structural commentary   

**Figure 1:**
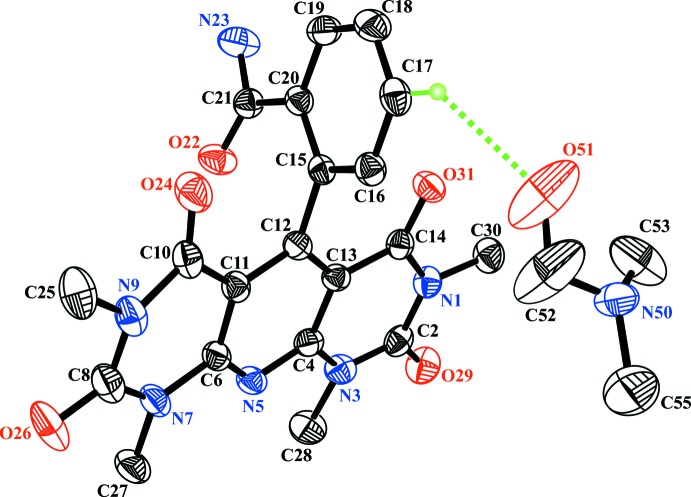
The mol­ecular structure with thermal displacement ellipsoids drawn at the 50% probability level (H atoms omitted for clarity).

The mol­ecular structure of the title compound is depicted in Fig. 1 ▶. The mol­ecule of (I) contains two almost planar fragments: (i) pyridodi­pyrimidine (r.m.s. deviation = 0.046 Å) and (ii) benzamide (r.m.s. deviation = 0.010 Å). The dihedral angle between them is 82.26 (11)°. The dimethyl formamide solvent mol­ecule is disordered about a crystallographic twofold axis.

## Supra­molecular features   

**Figure 2:**
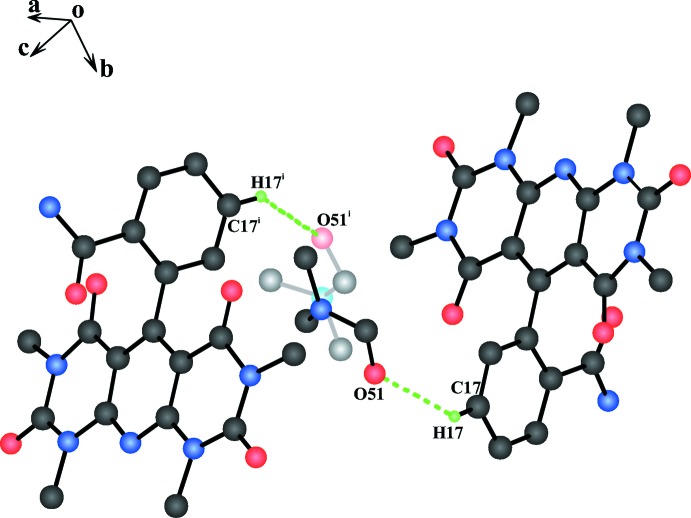
Non-classical hydrogen bonding of disordered DMFA solvent molecules with mol­ecules of 2-(1,3,7,9-tetra­methyl-2,4,6,8-tetra­oxo-1,2,3,4,6,7,8,9-octa­hydro­pyrido[2,3-*d*:6,5-*d*′]dipyrimidin-5-yl)benzamide related by the twofold axes of the space group.

**Figure 3:**
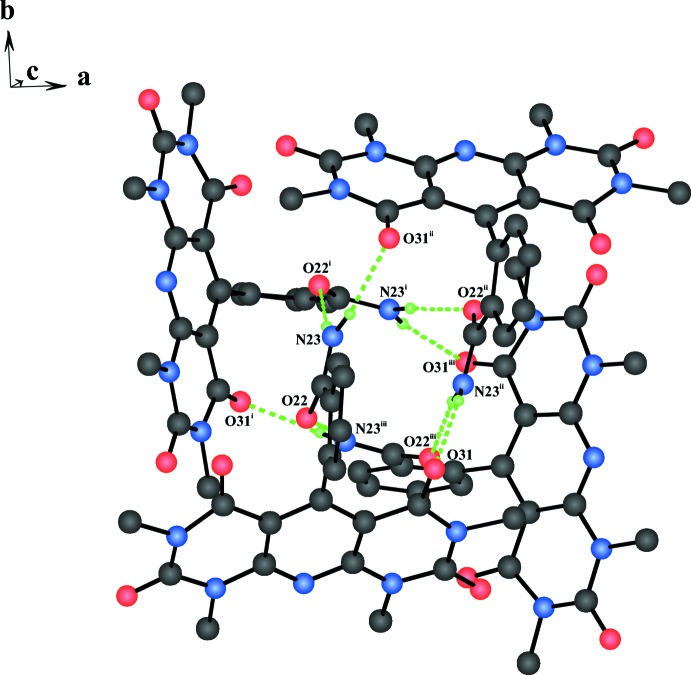
Square-prismatic telomer formed by four 2-(1,3,7,9-tetra­methyl-2,4,6,8-tetra­oxo-1,2,3,4,6,7,8,9-octa­hydro­pyrido[2,3-*d*:6,5-*d*′]dipyrimidin-5-yl)<0.02pt>benz­amide mol­ecules *via* inter­molecular hydrogen bonding.

Each disordered DMF solvent mol­ecule is connected to an adjacent 2-(1,3,7,9-tetra­methyl-2,4,6,8-tetra­oxo-1,2,3,4,6,7,8,9-octa­hydro­pyrido[2,3-*d*:6,5-*d*′]dipyrimidin-5-yl)benzamide mol­ecule, related by twofold axes, *via* a non-classical C17–H17⋯O51 hydrogen bond (see Fig. 2 ▶ and Table 1 ▶). The hydrogen atoms of the amide group are involved in the formation of inter­molecular N23–H23*B*⋯O31^i^ and N23–H32*A*⋯O22^ii^ hydrogen bonds, which link four mol­ecules of the title compound into a four-membered tetra­mer with an almost square-prismatic shape (see Fig. 3 ▶). In the extended structure, the inter­actions between these telomeres have solely van der Waals character. It appears that the almost square-prismatic shape of these tetra­mers is responsible for the unusual high symmetry of this structure (space group *I* 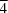
2*d*).

## Database survey   

In the Cambridge Structural Database, just three comparable structures were found: (i) 5-(4-fluoro­phen­yl)-1,3,7,9-tetra­methyl­pyrido[2,3-*d*:6,5-*d*]di­pyrimidine-2,4,6,8(1*H*,3*H*,7*H*,9*H*)-tetrone (Ghorbani & Bazgir, 2007 ▶); (ii) 5-(4-bromo­phen­yl)-1,3,7,9-tetra­methyl­pyrimido[5′,4′:5,6]pyrido[2,3-*d*]pyrimidine-2,4,6,8(1*H*,3*H*,7*H*,9*H*)-tetrone (Dabiri *et al.*, 2007 ▶); (iii) 1,3,7,9-tetra­methyl­pyrido[2,3-*d*:6,5-*d*′]di­pyrimidine-2,4,6,8-tetrone (Enrique-Miron *et al.*, 1994 ▶). The basic fragment for the title compound and compounds (i) and (ii) is the structure of compound (iii), in which the hydrogen atom of the pyridine ring is replaced by benzamide, fluoro­phenyl and bromo­phenyl respectively. There are no essential differences in the geometrical characteristics of corresponding chemical bonds, but the crystal packing of the mol­ecules differs essentially because of various features of the inter­molecular hydrogen bonding.

## Synthesis and crystallization   

A mixture of 6-amino-1,3-dimethyl-1,2,3,4-tetra­hydro-2,4-pyrimidine­dione 1.55 g (10 m*M*) and 1,2-benzene­dicarbonyl chloride 2.0 g (10 m*M*) dissolved in 10 ml DMFA was stirred under reflux for 2 h. The mixture was concentrated under reduced pressure, then 20 ml of iced water was added to it and filtered. The synthesized compound was dissolved in ethanol and crystallized by slow evaporation at room temperature (m.p. = 627–628 K, 55% yield).

## Refinement   

Crystal data, data collection details and structure refinement details are summarized in Table 2 ▶. The solvent mol­ecule of di­methyl­formamide is disordered about a crystallographic twofold axis. The coordinates of the H atoms of the phenyl ring and methyl groups were determined geometrically and refined using a riding model with the following restraints: for the phenyl ring, C—H = 0.93 Å, *U*_iso_(H) = 1.2*U*_eq_(C), and for the methyl groups, C—H = 0.96 Å, *U*_iso_(H) = 1.5*U*_eq_(C). Only the coordinates of the H atoms of the amide group, involved in hydrogen bonding, were determined from difference Fourier syntheses and refined freely.

## Supplementary Material

Crystal structure: contains datablock(s) global, I. DOI: 10.1107/S1600536814020200/hb7283sup1.cif

Structure factors: contains datablock(s) I. DOI: 10.1107/S1600536814020200/hb7283Isup2.hkl

Supporting information file. DOI: 10.1107/S1600536814020200/hb7283Isup3.cml

CCDC reference: 1023362

Additional supporting information:  crystallographic information; 3D view; checkCIF report

## Figures and Tables

**Table 1:** Hydrogen-bond geometry (Å, °)

*D*—H⋯*A*	*D*—H	H⋯*A*	*D*⋯*A*	*D*—H⋯*A*
N23—H23*B*⋯O31^i^	0.88 (3)	2.19 (3)	3.003 (4)	153 (3)
N23—H23*A*⋯O22^ii^	0.96 (4)	2.09 (4)	3.017 (4)	164 (3)
C17—H17⋯O51	0.93	2.56	3.313 (10)	138

Symmetry codes: (i) 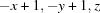; (ii) 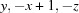.

**Table 2:** Experimental details

Crystal data	
Chemical formula	2C_20_H_18_N_6_O_5_·C_3_H_7_NO
*M*_r_	917.90
Crystal system, space group	Tetragonal, *I* 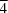 2*d*
Temperature (K)	293
*a*, *c* (Å)	26.173 (4), 12.434 (3)
*V* (Å^3^)	8517 (3)
*Z*	8
Radiation type	Mo *K*α
μ (mm^−1^)	0.11
Crystal size (mm)	0.16 (radius)

Data collection	
Diffractometer	Enraf–Nonius CAD-4
No. of measured, independent and observed [*I* > 2σ(*I*)] reflections	6734, 6216, 3247
*R*_int_	0.015
(sin θ/λ)_max_ (Å^−1^)	0.703

Refinement	
*R*[*F* ^2^ > 2σ(*F* ^2^)], *wR*(*F* ^2^), *S*	0.056, 0.135, 1.01
No. of reflections	6216
No. of parameters	339
No. of restraints	1
H-atom treatment	H atoms treated by a mixture of independent and constrained refinement
Δρ_max_, Δρ_min_ (e Å^−3^)	0.12, −0.17
Absolute structure	Flack *x* determined using 1083 quotients [(*I* ^+^)−(*I* ^−^)]/[(*I* ^+^)+(*I* ^−^)] (Parsons *et al.*, 2013 ▶)
Absolute structure parameter	0.0 (10)

Computer programs: *CAD-4 Software* (Enraf–Nonius, 1988 ▶), *HELENA* (Spek, 1997 ▶), *SHELXS2014* and *SHELXL2014* (Sheldrick, 2008 ▶), *ORTEP-3 for Windows* (Farrugia, 2012 ▶), *enCIFer* (Allen *et al.*, 2004 ▶) and *publCIF* (Westrip, 2010 ▶).

## supplementary crystallographic information

### Crystal data

**Table:**

2C_20_H_18_N_6_O_5_·C_3_H_7_NO	*D*_x_ = 1.432 Mg m^−^^3^
*M**_r_* = 917.90	Mo *K*α radiation, λ = 0.71073 Å
Tetragonal, *I*42*d*	Cell parameters from 24 reflections
*a* = 26.173 (4) Å	θ = 12.2–17.3°
*c* = 12.434 (3) Å	µ = 0.11 mm^−^^1^
*V* = 8517 (3) Å^3^	*T* = 293 K
*Z* = 8	Spherical, colourless
*F*(000) = 3840	0.16 × 0.16 × 0.16 × 0.16 (radius) mm

### Data collection

**Table:**

Enraf–Nonius CAD-4 diffractometer	*R*_int_ = 0.015
Radiation source: fine-focus sealed tube	θ_max_ = 30.0°, θ_min_ = 1.6°
Graphite monochromator	*h* = −26→26
θ/2θ scans	*k* = −36→36
6734 measured reflections	*l* = −17→17
6216 independent reflections	1 standard reflections every 60 min
3247 reflections with *I* > 2σ(*I*)	

### Refinement

**Table:**

Refinement on *F*^2^	Hydrogen site location: mixed
Least-squares matrix: full	H atoms treated by a mixture of independent and constrained refinement
*R*[*F*^2^ > 2σ(*F*^2^)] = 0.056	*w* = 1/[σ^2^(*F*_o_^2^) + (0.0536*P*)^2^ + 1.2893*P*] where *P* = (*F*_o_^2^ + 2*F*_c_^2^)/3
*wR*(*F*^2^) = 0.135	(Δ/σ)_max_ = 0.006
*S* = 1.01	Δρ_max_ = 0.12 e Å^−^^3^
6216 reflections	Δρ_min_ = −0.17 e Å^−^^3^
339 parameters	Absolute structure: Flack *x* determined using 1083 quotients [(*I*^+^)-(*I*^-^)]/[(*I*^+^)+(*I*^-^)] (Parsons *et al.*, 2013)
1 restraint	Absolute structure parameter: 0.0 (10)

### Special details

**Table:**

Geometry. All e.s.d.'s (except the e.s.d. in the dihedral angle between two l.s. planes) are estimated using the full covariance matrix. The cell e.s.d.'s are taken into account individually in the estimation of e.s.d.'s in distances, angles and torsion angles; correlations between e.s.d.'s in cell parameters are only used when they are defined by crystal symmetry. An approximate (isotropic) treatment of cell e.s.d.'s is used for estimating e.s.d.'s involving l.s. planes.

### Fractional atomic coordinates and isotropic or equivalent isotropic displacement parameters (Å^2^)

**Table:**

	*x*	*y*	*z*	*U*_iso_*/*U*_eq_	Occ. (<1)
N1	0.54916 (10)	0.33509 (10)	0.1396 (2)	0.0533 (7)	
C2	0.54598 (13)	0.30369 (12)	0.0506 (2)	0.0561 (8)	
N3	0.49790 (10)	0.29334 (9)	0.01248 (19)	0.0564 (7)	
C4	0.45404 (12)	0.31201 (11)	0.0602 (2)	0.0491 (7)	
N5	0.41001 (10)	0.29570 (9)	0.01939 (18)	0.0510 (6)	
C6	0.36754 (12)	0.31226 (11)	0.0642 (2)	0.0483 (7)	
N7	0.32283 (10)	0.29304 (10)	0.0228 (2)	0.0572 (7)	
C8	0.27519 (13)	0.30566 (13)	0.0635 (3)	0.0645 (10)	
N9	0.27443 (10)	0.33727 (10)	0.1518 (2)	0.0596 (8)	
C10	0.31609 (11)	0.36475 (13)	0.1894 (2)	0.0539 (8)	
C11	0.36577 (11)	0.34801 (11)	0.1489 (2)	0.0460 (7)	
C12	0.41230 (11)	0.36644 (10)	0.1882 (2)	0.0434 (6)	
C13	0.45766 (11)	0.34605 (10)	0.1470 (2)	0.0442 (7)	
C14	0.50830 (11)	0.35936 (11)	0.1884 (2)	0.0474 (7)	
C15	0.41236 (10)	0.40442 (10)	0.2778 (2)	0.0414 (6)	
C16	0.40476 (11)	0.38612 (11)	0.3818 (2)	0.0487 (7)	
H16	0.4010	0.3512	0.3931	0.058*	
C17	0.40282 (11)	0.41874 (12)	0.4672 (2)	0.0533 (8)	
H17	0.3967	0.4061	0.5359	0.064*	
C18	0.40994 (14)	0.47024 (12)	0.4523 (2)	0.0620 (9)	
H18	0.4094	0.4924	0.5107	0.074*	
C19	0.41787 (13)	0.48880 (11)	0.3495 (2)	0.0569 (8)	
H19	0.4227	0.5237	0.3395	0.068*	
C20	0.41875 (10)	0.45666 (10)	0.2610 (2)	0.0444 (7)	
C21	0.42413 (11)	0.47689 (11)	0.1484 (2)	0.0497 (8)	
O22	0.41550 (10)	0.45010 (8)	0.06982 (15)	0.0680 (6)	
N23	0.43726 (14)	0.52520 (11)	0.1365 (3)	0.0786 (10)	
H23A	0.4405 (14)	0.5378 (14)	0.065 (3)	0.088 (12)*	
H23B	0.4487 (13)	0.5433 (12)	0.191 (2)	0.069 (10)*	
O24	0.30973 (8)	0.39919 (10)	0.2537 (2)	0.0722 (7)	
C25	0.22403 (12)	0.35043 (16)	0.1963 (3)	0.0811 (12)	
H25A	0.2016	0.3216	0.1898	0.122*	
H25B	0.2101	0.3789	0.1573	0.122*	
H25C	0.2276	0.3594	0.2708	0.122*	
O26	0.23650 (9)	0.28828 (12)	0.0252 (2)	0.0939 (9)	
C27	0.32474 (15)	0.25925 (14)	−0.0717 (3)	0.0736 (11)	
H27A	0.3295	0.2794	−0.1354	0.110*	
H27B	0.2933	0.2406	−0.0772	0.110*	
H27C	0.3527	0.2357	−0.0642	0.110*	
C28	0.49437 (16)	0.26283 (14)	−0.0869 (3)	0.0777 (11)	
H28A	0.4900	0.2274	−0.0688	0.116*	
H28B	0.5251	0.2670	−0.1281	0.116*	
H28C	0.4657	0.2742	−0.1285	0.116*	
O29	0.58408 (9)	0.28574 (10)	0.00875 (19)	0.0769 (7)	
C30	0.60084 (12)	0.34458 (14)	0.1798 (3)	0.0697 (10)	
H30A	0.6183	0.3673	0.1317	0.105*	
H30B	0.6191	0.3129	0.1840	0.105*	
H30C	0.5990	0.3598	0.2499	0.105*	
O31	0.51616 (7)	0.38890 (9)	0.26314 (18)	0.0603 (6)	
N50	0.44874 (14)	0.25496 (10)	0.6322 (12)	0.0817 (17)	0.5
O51	0.4092 (3)	0.3226 (3)	0.6400 (9)	0.180 (4)	0.5
C52	0.3997 (5)	0.2778 (4)	0.6276 (15)	0.173 (6)	0.5
H52	0.3681	0.2622	0.6175	0.208*	0.5
C53	0.49626 (16)	0.2790 (3)	0.6091 (7)	0.126 (4)	0.5
H53A	0.4934	0.2979	0.5432	0.189*	0.5
H53B	0.5224	0.2534	0.6017	0.189*	0.5
H53C	0.5051	0.3018	0.6666	0.189*	0.5
C55	0.4465 (5)	0.20136 (14)	0.6134 (9)	0.141 (5)	0.5
H55A	0.4204	0.1942	0.5613	0.211*	0.5
H55B	0.4387	0.1840	0.6795	0.211*	0.5
H55C	0.4789	0.1897	0.5868	0.211*	0.5

### Atomic displacement parameters (Å^2^)

**Table:**

	*U*^11^	*U*^22^	*U*^33^	*U*^12^	*U*^13^	*U*^23^
N1	0.0482 (14)	0.0619 (15)	0.0498 (14)	0.0064 (12)	0.0030 (12)	0.0018 (13)
C2	0.0662 (19)	0.0569 (18)	0.0453 (16)	0.0124 (16)	0.0074 (16)	0.0080 (15)
N3	0.0700 (16)	0.0575 (14)	0.0416 (13)	0.0034 (13)	0.0066 (14)	−0.0056 (12)
C4	0.0633 (18)	0.0438 (15)	0.0403 (15)	0.0031 (14)	0.0002 (15)	0.0008 (13)
N5	0.0650 (15)	0.0483 (12)	0.0397 (12)	−0.0029 (12)	−0.0055 (13)	−0.0051 (11)
C6	0.0606 (18)	0.0444 (15)	0.0399 (14)	−0.0080 (14)	−0.0108 (14)	0.0073 (13)
N7	0.0667 (16)	0.0580 (15)	0.0470 (14)	−0.0150 (13)	−0.0187 (13)	0.0022 (13)
C8	0.065 (2)	0.073 (2)	0.0557 (19)	−0.0204 (17)	−0.0157 (17)	0.0133 (17)
N9	0.0501 (14)	0.0730 (17)	0.0556 (16)	−0.0119 (13)	−0.0073 (13)	0.0102 (14)
C10	0.0490 (16)	0.0661 (19)	0.0466 (16)	−0.0066 (15)	−0.0070 (14)	0.0078 (16)
C11	0.0494 (16)	0.0487 (15)	0.0400 (15)	−0.0037 (13)	−0.0047 (13)	0.0051 (13)
C12	0.0491 (14)	0.0422 (13)	0.0389 (13)	−0.0061 (13)	−0.0013 (13)	0.0039 (12)
C13	0.0495 (15)	0.0412 (14)	0.0419 (15)	−0.0025 (13)	0.0004 (13)	0.0006 (13)
C14	0.0473 (15)	0.0495 (16)	0.0453 (15)	−0.0001 (13)	0.0001 (14)	0.0056 (13)
C15	0.0364 (12)	0.0481 (14)	0.0398 (13)	0.0001 (13)	−0.0021 (12)	−0.0024 (12)
C16	0.0491 (16)	0.0520 (15)	0.0452 (14)	−0.0044 (14)	−0.0024 (14)	0.0036 (13)
C17	0.0516 (16)	0.0709 (19)	0.0375 (14)	0.0023 (16)	−0.0004 (13)	0.0013 (14)
C18	0.084 (2)	0.0601 (18)	0.0417 (15)	0.0050 (18)	−0.0008 (18)	−0.0108 (14)
C19	0.074 (2)	0.0454 (16)	0.0518 (16)	0.0043 (15)	−0.0009 (17)	−0.0045 (14)
C20	0.0427 (14)	0.0495 (15)	0.0409 (13)	0.0008 (13)	−0.0007 (13)	−0.0028 (13)
C21	0.0517 (17)	0.0484 (15)	0.0491 (17)	−0.0007 (13)	0.0010 (14)	0.0021 (14)
O22	0.1034 (17)	0.0591 (12)	0.0416 (11)	−0.0127 (13)	0.0011 (12)	−0.0043 (10)
N23	0.134 (3)	0.0529 (16)	0.0484 (16)	−0.0154 (17)	0.0014 (18)	0.0023 (14)
O24	0.0514 (12)	0.0916 (17)	0.0736 (15)	0.0044 (12)	−0.0026 (12)	−0.0222 (14)
C25	0.0446 (18)	0.118 (3)	0.080 (3)	−0.010 (2)	−0.0022 (18)	0.006 (2)
O26	0.0717 (15)	0.128 (2)	0.0816 (17)	−0.0388 (15)	−0.0298 (14)	−0.0001 (17)
C27	0.094 (3)	0.071 (2)	0.056 (2)	−0.013 (2)	−0.0233 (19)	−0.0092 (18)
C28	0.099 (3)	0.082 (2)	0.0515 (19)	0.010 (2)	0.007 (2)	−0.0215 (18)
O29	0.0739 (14)	0.0908 (16)	0.0659 (14)	0.0232 (14)	0.0178 (13)	−0.0051 (13)
C30	0.0494 (18)	0.083 (2)	0.076 (2)	0.0070 (16)	−0.0015 (17)	−0.003 (2)
O31	0.0492 (12)	0.0676 (13)	0.0642 (13)	−0.0020 (10)	−0.0057 (10)	−0.0177 (12)
N50	0.120 (4)	0.047 (3)	0.078 (4)	0.001 (5)	−0.009 (8)	−0.005 (3)
O51	0.164 (7)	0.124 (5)	0.252 (10)	0.065 (5)	0.057 (7)	0.065 (6)
C52	0.126 (9)	0.101 (7)	0.291 (17)	0.034 (7)	0.034 (11)	0.057 (10)
C53	0.149 (8)	0.140 (8)	0.089 (6)	−0.054 (7)	−0.002 (7)	−0.026 (7)
C55	0.193 (12)	0.091 (7)	0.138 (9)	−0.033 (8)	−0.025 (10)	0.031 (7)

### Geometric parameters (Å, º)

**Table:**

N1—C2	1.380 (4)	C18—H18	0.9300
N1—C14	1.384 (4)	C19—C20	1.385 (4)
N1—C30	1.463 (4)	C19—H19	0.9300
C2—O29	1.219 (4)	C20—C21	1.503 (4)
C2—N3	1.372 (4)	C21—O22	1.224 (3)
N3—C4	1.382 (4)	C21—N23	1.319 (4)
N3—C28	1.474 (4)	N23—H23A	0.96 (4)
C4—N5	1.330 (4)	N23—H23B	0.88 (3)
C4—C13	1.402 (4)	C25—H25A	0.9600
N5—C6	1.317 (4)	C25—H25B	0.9600
C6—N7	1.374 (4)	C25—H25C	0.9600
C6—C11	1.409 (4)	C27—H27A	0.9600
N7—C8	1.386 (4)	C27—H27B	0.9600
N7—C27	1.471 (4)	C27—H27C	0.9600
C8—O26	1.207 (4)	C28—H28A	0.9600
C8—N9	1.375 (4)	C28—H28B	0.9600
N9—C10	1.388 (4)	C28—H28C	0.9600
N9—C25	1.471 (4)	C30—H30A	0.9600
C10—O24	1.216 (4)	C30—H30B	0.9600
C10—C11	1.462 (4)	C30—H30C	0.9600
C11—C12	1.398 (4)	N50—C52	1.417 (13)
C12—C13	1.399 (4)	N50—C53	1.423 (5)
C12—C15	1.493 (4)	N50—C55	1.423 (5)
C13—C14	1.464 (4)	O51—C52	1.207 (14)
O31—C14	1.226 (3)	C52—H52	0.9300
C15—C16	1.393 (4)	C53—H53A	0.9600
C15—C20	1.393 (4)	C53—H53B	0.9600
C16—C17	1.364 (4)	C53—H53C	0.9600
C16—H16	0.9300	C55—H55A	0.9600
C17—C18	1.373 (4)	C55—H55B	0.9600
C17—H17	0.9300	C55—H55C	0.9600
C18—C19	1.383 (4)		
			
C2—N1—C14	125.3 (3)	C18—C19—H19	119.2
C2—N1—C30	115.5 (3)	C20—C19—H19	119.2
C14—N1—C30	119.1 (3)	C19—C20—C15	118.3 (3)
O29—C2—N3	121.8 (3)	C19—C20—C21	121.8 (3)
O29—C2—N1	121.5 (3)	C15—C20—C21	119.8 (2)
N3—C2—N1	116.7 (3)	O22—C21—N23	120.5 (3)
C2—N3—C4	122.9 (3)	O22—C21—C20	121.6 (3)
C2—N3—C28	117.0 (3)	N23—C21—C20	117.8 (3)
C4—N3—C28	120.0 (3)	C21—N23—H23A	117 (2)
N5—C4—N3	116.2 (3)	C21—N23—H23B	121 (2)
N5—C4—C13	123.8 (3)	H23A—N23—H23B	120 (3)
N3—C4—C13	119.9 (3)	N9—C25—H25A	109.5
C6—N5—C4	117.6 (2)	N9—C25—H25B	109.5
N5—C6—N7	116.1 (3)	H25A—C25—H25B	109.5
N5—C6—C11	124.2 (3)	N9—C25—H25C	109.5
N7—C6—C11	119.7 (3)	H25A—C25—H25C	109.5
C6—N7—C8	122.8 (3)	H25B—C25—H25C	109.5
C6—N7—C27	119.4 (3)	N7—C27—H27A	109.5
C8—N7—C27	117.7 (3)	N7—C27—H27B	109.5
O26—C8—N9	121.9 (3)	H27A—C27—H27B	109.5
O26—C8—N7	121.4 (3)	N7—C27—H27C	109.5
N9—C8—N7	116.6 (3)	H27A—C27—H27C	109.5
C8—N9—C10	124.7 (3)	H27B—C27—H27C	109.5
C8—N9—C25	117.0 (3)	N3—C28—H28A	109.5
C10—N9—C25	117.1 (3)	N3—C28—H28B	109.5
O24—C10—N9	119.9 (3)	H28A—C28—H28B	109.5
O24—C10—C11	124.8 (3)	N3—C28—H28C	109.5
N9—C10—C11	115.3 (3)	H28A—C28—H28C	109.5
C12—C11—C6	117.5 (3)	H28B—C28—H28C	109.5
C12—C11—C10	123.4 (3)	N1—C30—H30A	109.5
C6—C11—C10	119.1 (3)	N1—C30—H30B	109.5
C11—C12—C13	118.6 (2)	H30A—C30—H30B	109.5
C11—C12—C15	119.5 (3)	N1—C30—H30C	109.5
C13—C12—C15	121.8 (3)	H30A—C30—H30C	109.5
C12—C13—C4	117.8 (3)	H30B—C30—H30C	109.5
C12—C13—C14	123.3 (3)	C52—N50—C53	126.6 (7)
C4—C13—C14	118.9 (3)	C52—N50—C55	111.9 (9)
O31—C14—N1	119.5 (3)	C53—N50—C55	116.0 (8)
O31—C14—C13	124.7 (3)	O51—C52—N50	102.6 (11)
N1—C14—C13	115.8 (3)	O51—C52—H52	128.7
C16—C15—C20	119.6 (2)	N50—C52—H52	128.7
C16—C15—C12	117.6 (2)	N50—C53—H53A	109.5
C20—C15—C12	122.8 (2)	N50—C53—H53B	109.5
C17—C16—C15	120.9 (3)	H53A—C53—H53B	109.5
C17—C16—H16	119.6	N50—C53—H53C	109.5
C15—C16—H16	119.6	H53A—C53—H53C	109.5
C16—C17—C18	120.3 (3)	H53B—C53—H53C	109.5
C16—C17—H17	119.9	N50—C55—H55A	109.5
C18—C17—H17	119.9	N50—C55—H55B	109.5
C17—C18—C19	119.3 (3)	H55A—C55—H55B	109.5
C17—C18—H18	120.3	N50—C55—H55C	109.5
C19—C18—H18	120.3	H55A—C55—H55C	109.5
C18—C19—C20	121.6 (3)	H55B—C55—H55C	109.5
			
C14—N1—C2—O29	176.2 (3)	C10—C11—C12—C13	177.1 (3)
C30—N1—C2—O29	−0.6 (4)	C6—C11—C12—C15	180.0 (2)
C14—N1—C2—N3	−4.4 (4)	C10—C11—C12—C15	1.3 (4)
C30—N1—C2—N3	178.8 (3)	C11—C12—C13—C4	6.6 (4)
O29—C2—N3—C4	178.1 (3)	C15—C12—C13—C4	−177.8 (2)
N1—C2—N3—C4	−1.3 (4)	C11—C12—C13—C14	−174.8 (3)
O29—C2—N3—C28	−4.8 (4)	C15—C12—C13—C14	0.9 (4)
N1—C2—N3—C28	175.8 (3)	N5—C4—C13—C12	−4.2 (4)
C2—N3—C4—N5	−175.1 (3)	N3—C4—C13—C12	176.0 (3)
C28—N3—C4—N5	7.9 (4)	N5—C4—C13—C14	177.0 (3)
C2—N3—C4—C13	4.7 (4)	N3—C4—C13—C14	−2.7 (4)
C28—N3—C4—C13	−172.3 (3)	C2—N1—C14—O31	−175.5 (3)
N3—C4—N5—C6	179.1 (3)	C30—N1—C14—O31	1.2 (4)
C13—C4—N5—C6	−0.7 (4)	C2—N1—C14—C13	6.1 (4)
C4—N5—C6—N7	−177.5 (3)	C30—N1—C14—C13	−177.2 (3)
C4—N5—C6—C11	3.3 (4)	C12—C13—C14—O31	0.7 (5)
N5—C6—N7—C8	178.1 (3)	C4—C13—C14—O31	179.4 (3)
C11—C6—N7—C8	−2.7 (4)	C12—C13—C14—N1	179.0 (3)
N5—C6—N7—C27	−4.6 (4)	C4—C13—C14—N1	−2.3 (4)
C11—C6—N7—C27	174.7 (3)	C11—C12—C15—C16	79.1 (3)
C6—N7—C8—O26	179.8 (3)	C13—C12—C15—C16	−96.6 (3)
C27—N7—C8—O26	2.4 (5)	C11—C12—C15—C20	−99.8 (3)
C6—N7—C8—N9	−2.6 (4)	C13—C12—C15—C20	84.6 (4)
C27—N7—C8—N9	180.0 (3)	C20—C15—C16—C17	0.9 (4)
O26—C8—N9—C10	−169.4 (3)	C12—C15—C16—C17	−178.0 (3)
N7—C8—N9—C10	13.0 (5)	C15—C16—C17—C18	−2.0 (5)
O26—C8—N9—C25	−1.8 (5)	C16—C17—C18—C19	1.5 (5)
N7—C8—N9—C25	−179.4 (3)	C17—C18—C19—C20	0.1 (5)
C8—N9—C10—O24	165.2 (3)	C18—C19—C20—C15	−1.2 (5)
C25—N9—C10—O24	−2.4 (4)	C18—C19—C20—C21	176.2 (3)
C8—N9—C10—C11	−16.6 (4)	C16—C15—C20—C19	0.7 (4)
C25—N9—C10—C11	175.8 (3)	C12—C15—C20—C19	179.6 (3)
N5—C6—C11—C12	−0.8 (4)	C16—C15—C20—C21	−176.7 (3)
N7—C6—C11—C12	−180.0 (3)	C12—C15—C20—C21	2.1 (4)
N5—C6—C11—C10	177.9 (3)	C19—C20—C21—O22	−166.2 (3)
N7—C6—C11—C10	−1.3 (4)	C15—C20—C21—O22	11.1 (4)
O24—C10—C11—C12	6.9 (5)	C19—C20—C21—N23	12.0 (4)
N9—C10—C11—C12	−171.3 (3)	C15—C20—C21—N23	−170.6 (3)
O24—C10—C11—C6	−171.7 (3)	C53—N50—C52—O51	−25 (2)
N9—C10—C11—C6	10.1 (4)	C55—N50—C52—O51	−176.9 (13)
C6—C11—C12—C13	−4.3 (4)		

### Hydrogen-bond geometry (Å, º)

**Table:**

*D*—H···*A*	*D*—H	H···*A*	*D*···*A*	*D*—H···*A*
N23—H23*B*···O31^i^	0.88 (3)	2.19 (3)	3.003 (4)	153 (3)
N23—H23*A*···O22^ii^	0.96 (4)	2.09 (4)	3.017 (4)	164 (3)
C17—H17···O51	0.93	2.56	3.313 (10)	138

Symmetry codes: (i) −*x*+1, −*y*+1, *z*; (ii) *y*, −*x*+1, −*z*.
